# Idiopathic erythrocytosis as a potential early manifestation with IgA nephropathy: A case report and systematic review 

**DOI:** 10.5414/CNCS111797

**Published:** 2026-02-05

**Authors:** Yagmur Ersoy, Seyda Gul Ozcan, Mevlut Tamer Dincer, Ayse Salihoglu, Nurhan Seyahi, Sinan Trabulus

**Affiliations:** 1 Department of Internal Medicine,; 2 Division of Nephrology, and; 3 Division of Hematology, Department of Internal Medicine, Istanbul University-Cerrahpasa, Istanbul, Turkey

**Keywords:** IgA nephropathy, idiopathic erythrocytosis, review, glomerulonephritis

## Abstract

IgA nephropathy (IgAN) is the most common primary glomerulonephritis, characterized by mesangial IgA immune complex deposition, potentially leading to chronic kidney disease. Idiopathic erythrocytosis, an unexplained elevation of red blood cell mass, has rarely been reported in association with IgAN. We present a systematic literature review and a new case highlighting the temporal relationship between IgAN and idiopathic erythrocytosis. A PubMed search identified four relevant studies detailing IgAN accompanied by unexplained erythrocytosis. The literature suggests an association predominantly observed in male patients, typically concurrent with or subsequent to an IgAN diagnosis. In our case, however, idiopathic erythrocytosis was detected 2 years prior to the onset of clinical signs of IgAN. The patient, initially treated with phlebotomy, presented 2 years later with significant proteinuria, leading to a renal biopsy confirming IgAN. This temporal progression raises the hypothesis that polymeric IgA1 immune complexes may influence erythropoiesis prior to evident renal involvement. Clinicians should consider underlying glomerular disease in patients with unexplained erythrocytosis, even in the absence of overt renal symptoms.

## Introduction 

IgA nephropathy (IgAN) is the most prevalent primary glomerulonephritis worldwide, characterized by mesangial deposition of IgA immune complexes, leading to progressive renal dysfunction and, in some cases, secondary complications such as hypertension and hematuria [1]. 

Idiopathic erythrocytosis refers to an unexplained increase in red blood cell mass, often without a clear secondary cause, such as hypoxia, erythropoietin-secreting tumors, or polycythemia vera. Although erythrocytosis is commonly linked to myeloproliferative disorders, it has rarely also been reported in patients with IgAN [2]. Only a limited number of cases have described erythrocytosis in patients with IgAN. To enhance understanding of this association, we systematically reviewed the existing literature, focusing on the progression and temporal association between IgAN and erythrocytosis in previously reported cases to improve understanding of their relationship. Additionally, we present a recent case of a patient with idiopathic erythrocytosis preceding the diagnosis of IgAN. 

## Case report 

A 47-year-old male patient was referred to our clinic 2 years ago due to bilateral lower extremity edema. His medical history was notable for an incidental finding of elevated hemoglobin levels during routine blood testing at the age of 43. Initial investigations revealed negative results for JAK2, CALR, BCR-ABL, and MPL genetic mutations, excluding common myeloproliferative disorders. Bone marrow biopsy at that time demonstrated increased erythroid and granulocytic series, leading to a diagnosis of idiopathic erythrocytosis. The patient was managed with intermittent therapeutic phlebotomy to maintain hematocrit levels below 46% and daily acetylsalicylic acid. 

At presentation, physical examination showed mild bilateral lower extremity edema. Laboratory evaluation revealed mildly elevated serum creatinine (1.27 mg/dL), normal serum albumin (4.23 g/dL), and markedly elevated triglycerides (467 mg/dL). Urinalysis indicated significant proteinuria (dipstick protein +3), with rare leukocytes, no hematuria. Renal ultrasound demonstrated increased bilateral renal parenchymal echogenicity. The 24-hour urine analysis revealed a total protein level of 5,779.4 mg/day and 4,972 mg/day microalbumin. 

Renal biopsy findings confirmed IgA nephropathy accompanied by chronic interstitial nephritis and tubular injury (MEST classification: M1, E0, S1, T2). Additional serological investigations, including ANA, ANCA, dsDNA antibodies, complement components (C3, C4), and hepatitis screening, were negative. Further evaluation for erythrocytosis showed erythropoietin (EPO) levels at 3.7 mIU/mL (reference range: 4.9 – 29.0 mIU/mL), with no evidence of underlying pulmonary disease or history of tobacco use. 

Following the diagnosis, ramipril and methylprednisolone (1 mg/kg/day) therapy was initiated. Methylprednisolone was later discontinued due to hyperglycemia and recurrent urinary tract infections. The patient subsequently participated in a phase 3 clinical trial involving narsoplimab (3), but treatment was discontinued at week 12. He has been receiving budesonide (3 mg 3 times daily), dapagliflozin (10 mg daily), and ramipril (5 mg daily) for the last 9 months. Laboratory parameters during the clinical course of IgAN are detailed in Table 1. 

## Literature review 

We used the PubMed interface (pubmed.gov) to conduct a systematic literature review aimed at identifying studies that reported an association between idiopathic erythrocytosis and IgAN. The search was carried out in April 2025 using a combination of two keyword groups. The first group included “IgA nephropathy” and “Berger’s disease,” while the second group included “erythrocytosis,” “polycythemia,” and “idiopathic erythrocytosis.” Keywords within each group were combined using the logical operator “OR,” and the two groups were combined using the logical operator “AND.” 

The initial search yielded 14 articles. We excluded editorials, reviews, and unrelated studies. Additionally, articles focusing solely on polycythemia vera or secondary causes of erythrocytosis were excluded. After title and abstract screening, 10 articles were excluded due to irrelevance. The remaining 4 articles underwent full-text review to confirm inclusion eligibility. Following detailed evaluation, we identified 3 case reports and 1 cohort study that described a total of 9 individual patients with idiopathic erythrocytosis in the context of biopsy-proven IgAN. These cases, along with the present case, are summarized in Table 2. 

## Discussion 

Previous studies have reported an association between IgAN and polycythemia vera; however, only limited evidence exists regarding a potential relationship between idiopathic erythrocytosis and IgAN. A systematic review of available literature identified 3 detailed case reports and 1 cohort study describing this rare association [4, 5, 6]. Mahesh et al. [4] described a male patient who was diagnosed simultaneously with idiopathic erythrocytosis and IgAN, suggesting a possible pathogenic interaction between erythrocytosis and IgAN through shared immune-mediated mechanisms or erythropoietic dysregulation. Navaradnam et al. [5] similarly reported another male patient whose erythrocytosis diagnosis coincided with IgAN onset, proposing an immune-mediated mechanism involving IgA immune complex deposition potentially affecting erythropoiesis. Additionally, Lehmann et al. [6] presented a particularly unusual case highlighting the coexistence of immune thrombocytopenia, erythrocytosis, and IgAN in the same patient, further supporting the hypothesis that immune dysregulation in IgAN may trigger hematologic abnormalities beyond renal involvement. 

Our case provides an important addition to existing literature, illustrating a different temporal relationship. Unlike prior reports, our patient developed idiopathic erythrocytosis 2 years before any clinical evidence of renal disease. Common secondary causes were thoroughly excluded, including hypoxia, smoking, pulmonary or cardiac disease, erythropoietin-secreting tumors, and elevated EPO levels. Negative JAK2, CALR, MPL, and BCR-ABL mutation testing effectively ruled out myeloproliferative neoplasms. This timeline raises the possibility that erythrocytosis may precede renal involvement in some patients with IgAN. 

A recent study by Cohen et al. [7] provides important insight into the link between IgA nephropathy and erythrocytosis. The authors showed that polymeric and galactose-deficient IgA1 (Gd-IgA1) can increase the sensitivity of erythroid progenitor cells to EPO, promoting red blood cell production. In vitro experiments demonstrated enhanced erythroid colony formation, which diminished when IgA1 was removed. Similarly, transgenic mice expressing human IgA1 developed elevated hematocrit levels despite normal EPO concentrations. These findings suggest that circulating IgA1 may directly stimulate erythropoiesis, offering a potential explanation for erythrocytosis in IgAN, even before overt renal symptoms appear [7]. The clinical evolution of our patient, following IgAN-specific treatments, reduced erythrocytosis severity, and cessation of therapeutic phlebotomy, adds additional support to this hypothesis. 

In conclusion, clinicians should consider renal screening in patients with unexplained erythrocytosis, particularly in normal or low EPO levels and the absence of markers of myeloproliferative diseases. 

## Statement of ethics 

The present work was conducted in accordance with the Declaration of Helsinki. 

## Consent to publish 

Informed consent was obtained from the patient for publication of this case report. 

## Authors’ contributions 

Yagmur Ersoy collected patient data and contributed to drafting the manuscript. Seyda Gul Ozcan contributed to data interpretation and manuscript writing. Mevlut Tamer Dincer and Ayse Salihoglu participated in the critical revision of the manuscript. Nurhan Seyahi supervised the study, provided intellectual input, and contributed to manuscript preparation. Sinan Trabulus conceived and designed the study, coordinated data collection, and finalized the manuscript. 

## Funding 

None declared. 

## Conflict of interest 

The authors have no conflicts of interest to declare. 

**Table 1.** Laboratory parameters during the clinical course of IgA nephropathy.

**Parameter / timeline**	**At erythrocytosis diagnosis**	**At IgAN diagnosis**	**At the end of methylprednisolone treatment**	**At the end of the week of narsoplimab treatment**	**Current**
Creatinine (mg/dL)	1.04	1.27	1.32	1.49	1.2
Hemoglobin (g/dL)	20.03	14.5	13.4	15	14.4
Hematocrit (%)	60.89	43.1	40.3	45.1	45.7
Uric acid (mg/dL)	9.4	8.4	6.1	7.1	6.7
Total proteinuria (mg/day)	N/A	5,779.4	3,851.75	2,103.3	2,054.25
Microalbumin (mg/day)	N/A	4,972	3,429.66	1,629.34	1,531.2
Urine dipstick protein	Negative	3+	3+	3+	3+
Treatment	Allopurinol 150 mg daily, acetylsalicylic acid 100 mg daily, therapeutic phlebotomy	Allopurinol 150 mg daily, acetylsalicylic acid 100 mg daily, ramipril 5 mg daily	Allopurinol 150 mg daily, acetylsalicylic acid 100 mg daily, ramipril 5 mg daily, methylprednisolone 1 mg/kg daily	Allopurinol 150 mg daily, acetylsalicylic acid 100 mg daily, ramipril 5 mg daily, narsoplimab	Allopurinol 150 mg daily, acetylsalicylic acid 100 mg daily, ramipril 5 mg daily, dapagliflozin 10 mg daily, budesonid 9 mg a day

**Table 2.** Review of reported cases of IgA nephropathy associated with idiopathic erythrocytosis.

Reference no	First author (Year)	Age / Sex	Age at erythrocytosis diagnosis	Age at IgAN diagnosis	Initial Cr (mg/dL)	Initial 24h proteinuria (g/day)	EPO level	Bone marrow findings	Mutation	Treatment for IgAN	Renal outcome*
4	Mahesh (2017)	31 / M	31	31	2.20	7.5	Normal	Normal	JAK2 (-)	ACEi	Not applicable
5	Navaradnam (2021)	35 / M	35	35	3.19	Not applicable	Low	Erythroid hyperplasia with normal other cell lines	JAK (-), CALR (-) MPL (-)	ACEi, steroid, mycophenolate mofetil	Stage IIIa CKD
7	Cohen (2021)	64 / M	64	20	0.56	Negative	Normal	Not applicable	JAK (-)	ARB	Stage IIIb CKD
7	Cohen (2021)	74 / M	74	63	2.60	4	Normal	Not applicable	JAK (-)	ACEi and ARB	Stage IV CKD
7	Cohen (2021)	69 / M	69	26	0.99	2.3	Normal	Not applicable	JAK (-)	ACEi	Stage IIIa CKD
7	Cohen (2021)	45 / M	45	20	1.17	0.3	Normal	Not applicable	JAK (-)	ARB	Stage II CKD
7	Cohen (2021)	63 / M	61	44	1.33	0.4	Normal	Not applicable	JAK (-)	ARB	Stage IIIa CKD
7	Cohen (2021)	42 / M	26	21	2.53	2.3	Normal	Not applicable	JAK (-)	ARB	Stage IV CKD
6	Lehmann (2024)	36 / M	26	36	Not applicable	Not applicable	Normal-elevated	Erythroid hyperplasia with normal other cell lines	JAK (-)	Not applicable	Stage IIIb CKD
	Present case (current report)	47 / M	43	45	1.27	5.7	Low-normal	Increased erythroid and granulocytic series	JAK (-), CALR (-), MPL (-), BCR-ABL (-)	ACEi, steroid, narsoplimab, dapagliflozin, budesonide	Stage II CKD

ACEi = angiotensin-converting enzyme inhibitor; ARB = angiotensin receptor blocker; CKD = chronic kidney disease; *All stages are calculated by the CKD stage by CKD-EPI creatinine.

## References

[1] Hassler JR IgA nephropathy: A brief review. Semin Diagn Pathol. 2020; 37: 143–147. 32241578 10.1053/j.semdp.2020.03.001

[2] Aoun M Jadoul M Anders HJ Erythrocytosis and CKD: A Review American Journal of Kidney Diseases. 2024; 84: 495–506. 38621632 10.1053/j.ajkd.2024.02.015

[3] Lafayette R Rovin B Floege J Tesar V Zhang H Barratt J POS-132 TRIAL DESIGN: PHASE 3 RANDOMIZED, DOUBLE-BLIND, PLACEBO-CONTROLLED STUDY OF NARSOPLIMAB SAFETY AND EFFICACY IN IGA NEPHROPATHY (ARTEMIS-IGAN). Elsevier Kidney International Reports. 2022; 7: S57.

[4] Mahesh E Madhyastha PR Kalashetty M Gurudev KC Bande S John MM Idiopathic erythrocytosis in IgA nephropathy. Indian J Nephrol. 2017; 27: 72–73. 28182057 10.4103/0971-4065.194390 PMC5255996

[5] Navaradnam P Sujanitha V Suganthan N Sooriyakumar T Muthusamy M Immunoglobulin A Nephropathy with Erythrocytosis: An Unusual Association. Cureus. 2021; 13: e12437. 33763321 10.7759/cureus.13437 PMC7981785

[6] Lehmann P Bhatt UY Thakkar A A Unique Case of IgA Nephropathy Associated with Both Immune Thrombocytopenia and Erythrocytosis: TH-PO699. JASN. 2024; 35.

[7] Cohen C, et al. Erythrocytosis associated with IgA nephropathy. eBioMedicine 2021; 75: 103785. https://doi.org/10.1016/j.ebiom.2021.103785. 34959131 10.1016/j.ebiom.2021.103785 PMC8718985

